# Terahertz Spectral Properties of 5-Substituted Uracils

**DOI:** 10.3390/s21248292

**Published:** 2021-12-11

**Authors:** Kaixuan Li, Ding Li, Yan Zhang

**Affiliations:** 1 Beijing Advanced Innovation Center for Imaging Theory and Technology, Beijing Key Laboratory of Metamaterials and Devices, Key Laboratory of Terahertz Optoelectronics, Ministry of Education, Department of Physics, Capital Normal University, Beijing 100048, China; 18355630609@163.com (K.L.); lidingcnu@163.com (D.L.); 2 Beijing Key Laboratory of Metamaterials and Devices, Key Laboratory of Terahertz Optoelectronics, Ministry of Education, Beijing Advanced Innovation Center for Imaging Theory and Technology, Department of Physics, Capital Normal University, Beijing 100048, China

**Keywords:** terahertz time domain spectroscopy, absorption peaks, 5-substituents of uracil, vibration, broad spectrum, molecular structures

## Abstract

Applications of terahertz time-domain spectroscopy (THz-TDS) in the fields of chemistry and biomedicine have recently received increased attention. Specifically, THz-TDS is particularly effective for the identification of alkaloid molecules, because it can distinguish the vibration types of base molecules in the THz band and provide a direct characteristic spectrum for the configuration and conformation of biomolecules. However, when THz-TDS technology is used to identify alkaloid molecules, most of them are concentrated in the 0.1–3.0 THz band, limiting the amount of information that can be obtained. In this work, a wide-spectrum THz-TDS system was independently built to explore the absorption spectra of uracil and its 5-substituents in the range of 1.3–6.0 THz. We found that, in the THz band, uracil and its 5-substituents have similar absorption peaks near 4.9 and 3.3 THz, while the 5-substituents have an additional absorption peak in the range of 1.5–2.5 THz. This absorption peak is red-shifted as the relative atomic mass of the 5-substituted atoms increases. Gaussian software was adopted to calculate the absorption spectra of the samples. The simulation conclusions were in good agreement with the experimental results, revealing that the vibration of the base molecule at low frequencies can be attributed to the inter-molecular vibration. This work demonstrates that THz-TDS technology can be used to accurately identify biomolecules with similar molecular structures, reflecting the importance of molecular structure in biological activity.

## 1. Introduction

The terahertz (THz) band consists of electromagnetic waves located between infrared waves and microwaves on the electromagnetic spectrum. Its frequency range is usually defined as 0.1–10.0 THz. In recent years, due to the rapid development of ultra-fast laser technology and new materials, related technologies, such as THz sources and THz detectors [1,2,3,4], have been rapidly developed and improved, and the related applications have gradually entered a new stage. Due to its strong penetration, coherence, transient performance, non-destructive features, and information richness, THz radiation has been widely used in wireless communications, national defense and security, biomedicine, and other fields [5,6,7,8]. THz technology has come to provide important analytical methods in chemistry and pharmaceutical analysis. The low-frequency resonance absorption of biological macromolecules in the THz range has also received more and more attention in recent years. For biological macromolecules (e.g., nucleic acids, proteins, carbohydrates, and lipids), the absorption frequencies corresponding to the inter-/intra-molecular weak interaction forces (e.g., hydrogen bond, Van der Waals force, and so on) and the lattice vibrations are distributed in the THz frequency range [9,10,11,12,13,14]. Generally, the overall conformation of a molecule is important for large biological molecules (e.g., DNA and RNA), and it also plays an important role in the biological activity of small molecules (e.g., vitamins and alkaloid molecules [15,16,17].). Therefore, the unique characteristics of THz technology have been applied in biomedical research [18,19,20,21,22,23,24], and the connection between low-frequency vibration modes and molecular structure makes the spectroscopy of biomolecules quite interesting.

Uracil and its derivatives play a key role in basic biological processes. After researchers discovered that certain tumors preferentially incorporate uracil, rather than thymine into DNA, halogenated pyrimidines were synthesized in the 1950s as anti-tumor drugs [25]. The conversion of uracil to 5-halogen-uracil significantly changes its chemical and spectral characteristics and activity. Similarly, it has been found that uracil conversion products play an important role in a variety of micro-organisms and mammalian preparations and, as such, they can be used as anti-tumor, anti-bacterial, and anti-viral drugs. At present, their biological activity mechanisms are not fully understood; thus, further research on uracil and its substitutes is still needed. THz-TDS provides a powerful tool to carry out this type of research. However, the spectral range of conventional THz-TDS is usually concentrated below 3.0 THz [12,13,26,27,28] and information at higher frequencies is rarely reported, while biomolecules have abundant information at higher frequencies. In this article, we use an organic crystal—DAST (4-N,N-dimethylamino-4’-N’-methyl stilbazolium tosylate)—as a THz source to explore the absorption characteristics of 5-substituents of uracil in the range of 1.3–6.0 THz. The absorption spectra of these materials are also calculated using Gaussian software. Uracil and its four 5-substituents have great similarities in their structure. The main structure is a pyrimidine ring, and the positions of the 5-substituents are all those of halogen atoms with different relative atomic masses, which is convenient for analysis. The experiment and simulation results prove that uracil and its 5-substituents have similar absorption peaks near 4.9 and 3.3 THz, while the 5-substituents have one more absorption peak in the range of 1.5–2.5 THz. The absorption peak in the low-frequency range is red-shifted as the relative atomic mass of the 5-substituted atoms increases. We show that, in the molecular structure of biological samples, changes in substituents and differences in relative atomic mass have a particularly large impact on their properties and functions. This work provides a good demonstration of the reliability of THz-TDS technology in the field of biomedicine.

## 2. Materials and Methods

**Figure 1:**
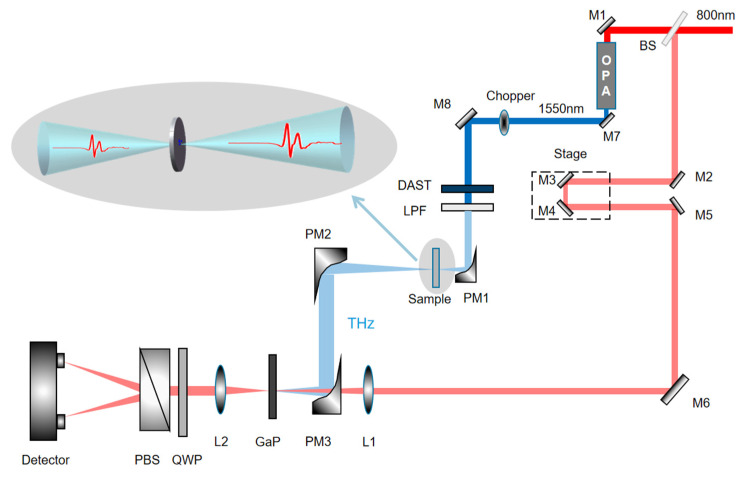
Experimental setup. BS: Beam splitter; PM: parabolic mirror; PBS: polarizing beam splitter, L: lens; M: mirror; LPF: low-pass filter; QWP: quarter wave plate.

**Figure 2:**
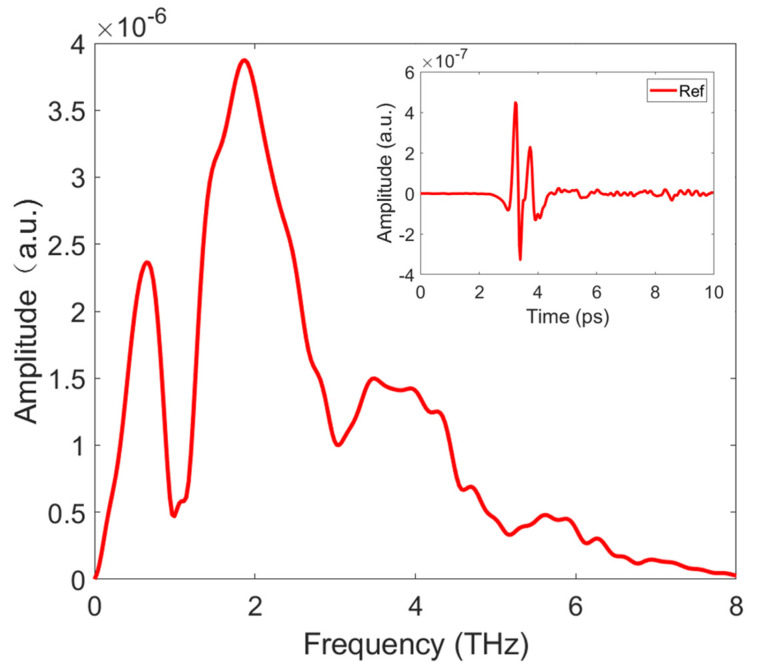
Terahertz reference signal. The effective spectral width is 0.1–8.0 THz, and the dip at 1.0 THz is caused by the absorption of the DAST crystal itself. The insert is a time-domain signal with a signal-to-noise ratio of 849.

The optical system used in the experiment was a home-built wide-band TDS. As shown in Figure 1, a beam splitter (BS) is used to divide 800 nm femtosecond light into two beams. The choice of BS with a splitting ratio of 98:2 solves the problems of the low main signal strength, low signal-to-noise ratio, and large echo signals caused by the previous BS with a splitting ratio of 90:10. The transmitted beam is used to pump an optical parametric amplifier, and the emitted 1550 nm light is employed to pump a DAST crystal (Qingdao University, Qingdao, China) for THz generation [29,30,31]. The detection crystal is a 200 mm-thick GaP crystal (Chenfang Optoelectronic Technology Co., Ltd, Hebei, China), which ensures that the high frequency of THz radiation can be measured. The THz time-domain signal of the system and its corresponding spectrum are shown in Figure 2. The spectral resolution is 0.1 THz and the signal-to-noise ratio is 849. The resolution of our system is not so high because the detection crystal used in our system is only 200 µm, which makes the required detection time window small and generates corresponding echoes, which eventually lead to a relatively low spectrum resolution. However, due to the relative wide absorption peaks of the samples we used, there are no issues when testing the samples with this resolution. It can be found that the effective spectrum of the system can reach 8.0 THz. The dip around 1.0 THz is caused by the absorption of the DAST crystal. In order to reduce the influence of water vapor, the experiment was conducted in a closed chamber filled with dry air.

**Figure 3:**
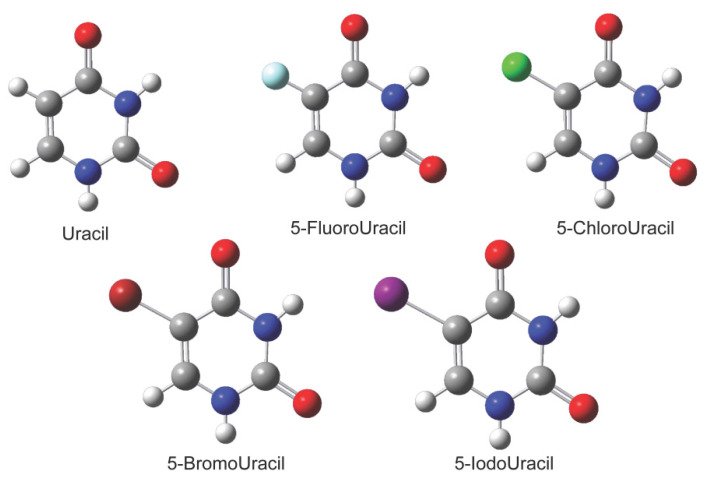
Molecular structures of uracil and 5-substituted-uracils. The 5-substitution atoms are F, Cl, Br, and I, respectively.

The samples selected were uracil and four types of its 5-substituents, namely, 5-Fluorouracil, 5-Chlorouracil, 5-Bromouracil, and 5-Iodouracil. The substituted atoms are halogen atoms of the seventh group, with relative atomic masses of 18.9, 35.5, 79.9, and 127.0, respectively. The chemical structure diagrams of the samples are shown in Figure 3. Uracil and its 5-substituents have pyrimidine ring structures, which are mainly connected to carbon, hydrogen, and oxygen atoms with single or double bonds. The samples were purchased from Aladdin Reagent Company, Shanghai, China, with purity up to 99%. The samples were ground into a fine powder with a mortar and pestle and mixed with polyethylene powder at a ratio of 1:10. A total of 120 mg of each of the mixed samples were pressed into discs with a thickness of 0.7–0.9 mm and a diameter of 13.0 mm.

## 3. Results

### 3.1. Experimental Results

**Figure 4:**
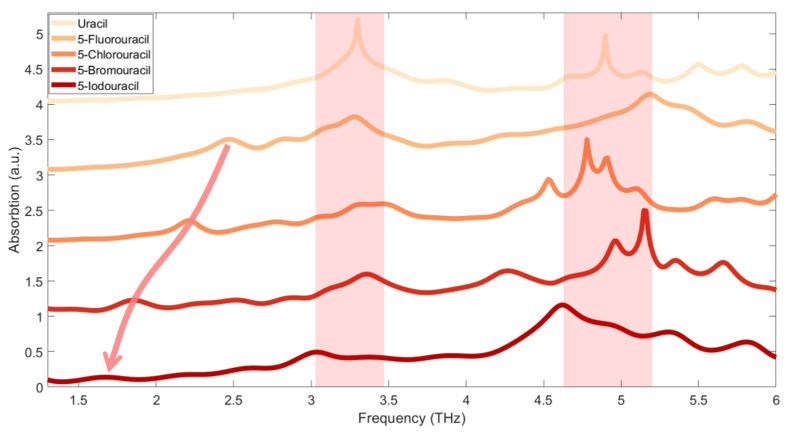
Molecular absorption spectra measured in the experiment. The red shaded areas near 3.3 and 4.9 THz indicate the fluctuation range of the absorption peaks shared by the five samples. The red arrow indicates the direction in which the red-shift of the additional absorption peak for the sample’s low-frequency absorption peaks moves the 5-substituted samples.

The experimentally measured absorption spectra of the samples are shown in Figure 4. The spectra are shifted perpendicularly for clarity. It can be seen that uracil has two absorption peaks, obviously located around 4.9 and 3.3 THz, and its 5-substituents (i.e., 5-Fluorouracil, 5-Chlorouracil, 5-Bromouracil, and 5-Iodouracil) also have absorption peaks around 4.9 and 3.3 THz. However, in contrast to uracil, the four 5-substituents have another absorption peak below 2.5 THz. Furthermore, it can also be seen that this absorption peak is red-shifted as the relative atomic mass of the 5-substituted atom increases. The absorption peaks near 3.3 and 4.9 THz are caused by the mutual expansion and contraction of the atomic bonds on the pyrimidine ring, and the vibration modes play a key role. When the fifth position of uracil is replaced by a halogen atom, the relative atomic mass of the whole molecule increases, and the position of the center of mass is also shifted relative to uracil. At the same time, the halogen atom interacts with surrounding molecules, resulting in the absorption peaks of 5-substituted-uracils shifting and broadening relative to uracil, at 4.9 THz and 3.3 THz. However, the 5-substituted atoms participate and play a key role in the vibration corresponding to the absorption peak around 2.5 THz. When the relative atomic mass of the 5-substituted atom becomes larger, molecular vibration becomes relatively difficult; therefore, there is a red-shift of the absorption peaks.

### 3.2. Simulation Results

**Figure 5:**
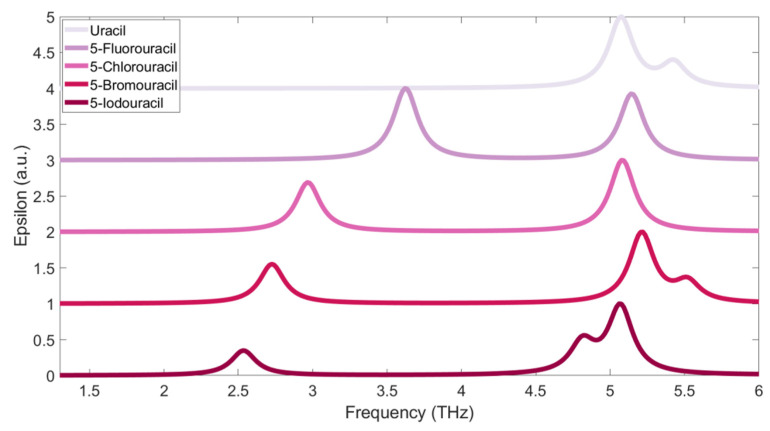
Simulation results of uracil and its 5-substitutions using Gaussian software.

In order to confirm the conjecture about the experimental results, the Gaussian 09w software package was used to explain the weak interaction of the samples in the THz region [22]. The structures of the samples were optimized and the corresponding absorption spectra were calculated. For uracil, 5-Fluorouracil, 5-Chlorouracil, and 5-Bromouracil, B3LYP/6-311++g was chosen as the hybrid function and bases set, whereas B3LYP/LANL2DZ was chosen for 5-Iodouracil. The simulation results are shown in Figure 5. Uracil and all of its four 5-substituents have an absorption peak near 5.0 THz, located at 5.07, 5.14, 5.07, 5.20, and 5.07 THz, respectively. At the same time, the four 5-substituents present an absorption peak that uracil does not have in the low-frequency range. Furthermore, this absorption peak red-shifts when the relative atomic mass of the 5-substituted atom increases. Hence, the simulation results were in agreement with the experimental data.

**Figure 6:**
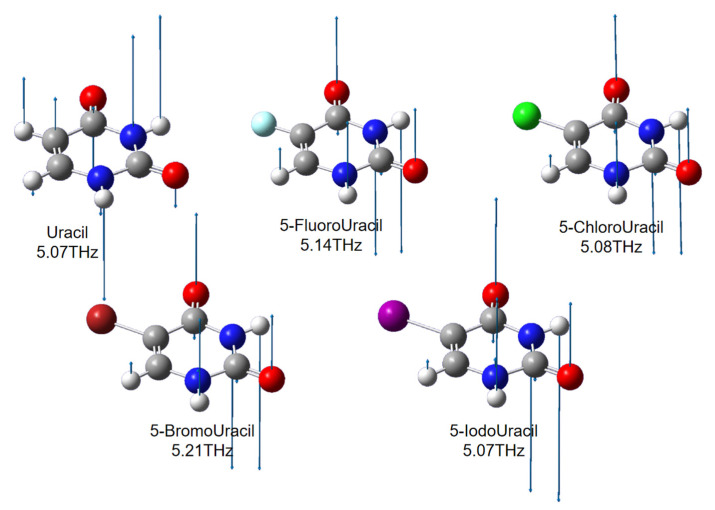
Vibration vector diagrams of the absorption peak for all samples near 5.0 THz. The arrow indicates the vibration direction of the atom, and the length of the line segment indicates the amplitude.

In order to identify the absorption peaks, the vibration vector diagrams corresponding to the absorption peaks for five molecules are presented. The vibration vector diagrams of the absorption peaks of all five samples around 5.0 THz are shown in Figure 6, and the corresponding dynamic diagrams are provided in the supplementary literature (Please see Videos S1–S9). From Figure 6, it can be seen that the vibration forms of all four 5-substituents were almost the same, with the atoms on the pyrimidine ring oscillating in the direction perpendicular to the ring. Due to the large relative atomic mass of the four substituents and the strong electronegativity, uracil and its 5-substituents present different vibration forms. This phenomenon verified the conjecture regarding the experimental results. 

**Figure 7:**
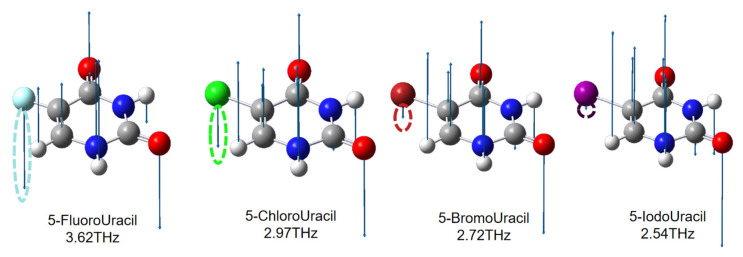
Vibration vector diagrams of the absorption peaks in the low-frequency range. The arrow indicates the vibration direction of the atom, and the length of the line segment indicates the amplitude. The vibration of the substituted atom is indicated by the dotted ellipse with corresponding color.

For comparison, the vibration vector diagrams for the absorption peaks in the low-frequency range for the four 5-substituents are shown in Figure 7. It can be seen that the substituents participate in the molecular vibration process, swinging perpendicular to the direction of the pyrimidine ring. This also explains why uracil has no absorption peak at low frequencies. When the relative atomic mass of the 5-substituted atom increases, this vibration becomes difficult and, thus, the vibration moves to a lower frequency. This demonstrates that THz-TDS combined with the Gaussian software can accurately identify biomolecules with similar structures.

## 4. Discussion

Although the simulation results were in agreement with the experimental data, there was a certain difference between them. All the measured results had absorption peaks near 4.9 and 3.3 THz, while the simulation only had a common absorption peak near 5.0 THz. Furthermore, the measured data were blue-shifted relative to the simulation results. In the experiment, the samples were in crystal states. When the biomolecules are stacked in crystals within a close range, they can interact with each other, usually produce hydrogen bonds that are weaker than the covalent bonds and ionic bonds, and the mass of motion involved is very large. Therefore, the resonance frequency of the vibration mode dominated by hydrogen bonds is lower than the typical intra-molecular resonance frequency and falls within a lower frequency range. The simulation only considered a single molecule in the gaseous state and ignored the interactions between molecules. This is why the peak at 3.3 THz did not appear in the simulation. The experiment was carried out at room temperature (297 K), and the simulation assumed that the sample was at an ideal temperature of 0 K. Furthermore, the grinding time, the mixing ratio of polyethylene, and the uniformity of the tablet also affected the results of the experiment to some extent. 

## 5. Conclusions

The absorption spectra of uracil and four of its 5-substituents in the range of 1.3–6.0 THz were measured using a home-built THz-TDS, with a spectrum width of 0.1–8.0 THz. The measured data showed that the five samples had similar absorption peaks near 4.9 and 3.3 THz, mainly due to the absorption caused by mutual stretching and swinging between atoms on the pyrimidine ring of the sample. The swing forms of the molecule structures were almost the same. The presence of 5-sustituent atoms leads to a new vibration mode, which causes an absorption peak in the low-frequency range. The absorption peak was observed to red-shift with an increase in the relative atomic mass of the 5-substituted atom. Simulation results achieved using Gaussian software matched the experimental data well. The difference between the experimental and simulation results was mainly caused by the fact that a single molecule structure was used in the simulation, while a multi-molecular crystal was measured in the experiment. Another difference is that the experiment was performed at room temperature, while the simulation was assumed to be at 0 K. This work demonstrates that the THz-TDS combined with the Gaussian software can provide an effective approach for understanding and identifying molecular structures for biological and biomedical applications.

## Data Availability

The data that support the findings of this study are available from the corresponding author upon reasonable request.

## Supplementary Materials

The following are available online at https://www.mdpi.com/article/10.3390/s21248292/s1: Video S1: Dynamic diagrams of Uracil at 5.07 THz; Video S2: Dynamic diagrams of 5-Fluorouracil at 5.14 THz; Video S3: Dynamic diagrams of 5-Chlorouracil at 5.08 THz; Video S4: Dynamic diagrams of 5-Bromouracil at 5.21 THz; Video S5: Dynamic diagrams of 5-Iodouracil at 5.06 THz; Video S6: Dynamic diagrams of 5-Fluorouracil at 3.62 THz; Video S7: Dynamic diagrams of 5-Chlorouracil at 2.97 THz; Video S8: Dynamic diagrams of 5-Bromouracil at 2.73 THz; Video S9: Dynamic diagrams of 5-Iodouracil at 2.53 THz.
